# Comparative Recovery After Acute Lower-Limb Wounds Treated with Negative-Pressure Wound Therapy and Three Gradations of Manual Rehabilitation

**DOI:** 10.3390/healthcare13131496

**Published:** 2025-06-23

**Authors:** Cristina-Teodora Stanciu, Milan Daniel Velimirovici, Dinu Vermesan, Ciprian Nicolae Pilut, Loredana Stana, Felix Bratosin, Daniel Laurentiu Pop, Bogdan Hogea

**Affiliations:** 1 Doctoral School, “Victor Babes” University of Medicine and Pharmacy, 300041 Timisoara, Romania; cristina.stanciu@umft.ro; 2 Department I Nursing, Faculty of Nursing, “Victor Babes” University of Medicine and Pharmacy, 300041 Timisoara, Romania; milan.velimirovici@umft.ro; 3 Department XVI—Orthopedics, Traumatology, Urology, and Medical Imaging, Discipline of Orthopedics and Traumatology I, “Victor Babes” University of Medicine and Pharmacy, 300041 Timisoara, Romania; dinu@vermesan.ro (D.V.); daniellaurentiupop@yahoo.com (D.L.P.); hogea.bogdan@umft.ro (B.H.); 4 Department of Dermatology, “Victor Babes” University of Medicine and Pharmacy, 300041 Timisoara, Romania; 5 Department I, Discipline of Anatomy and Embryology, “Victor Babes” University of Medicine and Pharmacy, Eftimie Murgu Square 2, 300041 Timisoara, Romania; 6 Department of Infectious Diseases, “Victor Babes” University of Medicine and Pharmacy, Eftimie Murgu Square 2, 300041 Timisoara, Romania; felix.bratosin@umft.ro

**Keywords:** negative-pressure wound therapy, physical therapy modalities, proprioceptive neuromuscular facilitation, lower extremity injuries, wound healing

## Abstract

Background and Objectives: Negative-pressure wound therapy (NPWT) expedites tissue repair, yet functional recovery depends on adjunct rehabilitation. Evidence from high-resource settings is difficult to translate to Romanian county hospitals, where advanced devices are scarce. The objective of this study is to determine whether two tiers of low-technology, therapist-delivered exercise improve mobility, oedema resolution, pain and quality-of-life (QoL) beyond NPWT alone in adults with acute lower-limb wounds. Methods: A single-centre, prospective observational study (January 2021–June 2024) enrolled 92 patients and randomised them unevenly into: Group A, NPWT only (n = 39); Group B, NPWT + routine physiotherapy (n = 33); Group C, NPWT + enhanced manual programme (n = 20). All received −125 mmHg continuous suction; rehabilitation started 48 h post-operation. Primary outcomes were ankle dorsiflexion and knee flexion at 12 weeks. Secondary outcomes included calf circumference, ultrasound oedema depth, Manual Muscle Testing (MMT), pain (VAS), analgesic use and SF-36 domains through 24 weeks. Results: Baseline characteristics were similar (*p* > 0.40). At 12 weeks dorsiflexion reached 20.1 ± 1.8° in Group C, surpassing Group B (18.4 ± 2.1°; *p* = 0.004) and Group A (16.0 ± 2.3°; *p* < 0.001). Knee flexion followed the same gradient (140.8 ± 3.2°, 137.6 ± 3.4°, 133.4 ± 3.8° respectively). Oedema depth fell fastest in Group C (0.4 ± 0.2 mm by day 42) versus B (0.6 ± 0.2 mm) and A (0.8 ± 0.3 mm). Week-12 MMT grade ≥ 4.5 was attained by 95% of Group C, 85% of B and 72% of A (χ^2^ = 10.9, *p* = 0.004). VAS pain fell more steeply with each rehabilitation layer, paralleled by a stepwise decline in daily tramadol. All SF-36 domains were highest in Group C at 24 weeks (Physical Function 88.7 ± 4.8 vs. 85.1 ± 5.4 vs. 78.2 ± 5.9; *p* < 0.001). Mobility correlated positively with QoL (r = 0.66) and inversely with pain and oedema. Conclusions: In a resource-constrained Romanian setting, adding structured manual physiotherapy to NPWT produced meaningful functional and patient-centred gains, while an “enhanced” programme incorporating daily PNF and elastic-band strengthening delivered the largest observed benefit. These findings justify prioritising therapist-led interventions even where sophisticated equipment is unavailable.

## 1. Introduction

Acute wounds resulting from severe trauma or complex surgical interventions in the crural region continue to challenge healthcare providers. Although advances in surgical techniques and wound care materials have significantly improved patient prognosis, the restoration of complete functionality—encompassing joint mobility, muscle strength, and the capacity to resume everyday tasks—remains crucial [1,2]. In this context, negative-pressure wound therapy (NPWT) has emerged as a leading intervention for enhancing wound closure, reducing local inflammation, and preventing infectious complications [3]. Nevertheless, NPWT alone does not correct underlying neuromuscular deficits or impairments in proprioception, both of which are critical for a successful return to optimal physical activity [4].

Classical physiotherapy techniques, such as passive and active mobilizations, strengthening exercises, and muscle re-education routines, have long been recognized for their effectiveness in maintaining and improving the range of motion (ROM) and preventing muscle atrophy [5,6]. More specialized physiotherapy methodologies, including proprioceptive neuromuscular facilitation (PNF), Kabat diagonals, and manual lymphatic drainage, have been investigated for their potential to accelerate functional recovery by enhancing neuromuscular coordination, stimulating muscle activation, and alleviating oedema [7,8]. Physical therapy plays a crucial role in wound healing and oedema reduction by improving blood circulation and promoting lymphatic drainage, which are essential for tissue repair and fluid balance. Classical physiotherapy techniques such as mobilizations and strengthening exercises enhance joint mobility and muscle function, supporting overall physical recovery.

Despite the broad clinical application of these interventions, robust comparative data specifically evaluating the combination of NPWT with classical physiotherapy versus NPWT integrated with specialized physiotherapy in acute lower-limb wounds are notably scarce [9]. Existing literature often focuses either on the benefits of NPWT in wound management or on general physiotherapy techniques in musculoskeletal rehabilitation, without detailing their joint contribution to outcomes such as joint mobility, muscle strength, oedema reduction, and overall quality of life across both short-term and extended follow-up [10,11].

We hypothesize that the inclusion of specialized physiotherapy techniques may result in earlier and more sustained improvements in functional outcomes, alongside comparable or slightly superior effects on oedema reduction. Furthermore, we anticipate that patients may experience more rapid pain relief and improved psychological well-being in the short term, with potential convergence of outcomes between groups at the 6-month mark [12]. The present work therefore pioneers a pragmatic, three-tier ‘dose–response’ model in which progressively richer, therapist-led but equipment-free interventions are layered onto standard NPWT. We hypothesised a stepwise improvement in biomechanics, oedema clearance, and patient-centred metrics with each additional rehabilitation tier, providing an implementation roadmap for hospitals that lack capital-intensive devices. No study has yet tested whether stepwise additions of therapist-delivered, low-technology exercise on top of standard NPWT yield a proportional ‘dose–response’ in biomechanics, swelling and patient-centred outcomes. Addressing this gap is crucial for hospitals that lack costly robotic or electro-mechanical devices but can invest staff time. We therefore designed a prospective study to test whether layering routine or “enhanced” low-technology rehabilitation onto NPWT improves biomechanics, oedema kinetics, pain control, analgesic consumption, and health-related QoL over six months, as well as to examine inter-relationships among biomechanical, oedema, and patient-reported outcomes.

## 2. Materials and Methods

### 2.1. Study Design and Ethical Compliance

This prospective observational study was undertaken at the SCJUPBT, Department of Orthopedics and Traumatology, affiliated with the “Victor Babeș” University of Medicine and Pharmacy, Timișoara. The recruitment period ranged from September 2020 to November 2024. Patients were evaluated at 6 weeks, 12 weeks, and 24 weeks, as well as measurements for oedema and calf girth evolution at 10 days, 6 weeks, and 6 months. A 2:2:1 ratio was chosen because therapist capacity exceeded pump availability; unequal allocation preserved external validity while keeping total sample feasible. There was no randomisation.

Electronic medical records were examined to retrieve demographic and clinical information, while direct evaluations were conducted to gather detailed functional and quality of life data. Patient confidentiality was maintained through secure data management protocols in strict accordance with international regulations, including the EU GCP Directive 2005/28/EC and the ethical principles outlined in the Declaration of Helsinki [12,13,14]. All participants provided written informed consent as mandated by national legal requirements (Article 167 of Law No. 95/2006 and Order 904/2006, Article 28, Chapter VIII).

### 2.2. Setting and Standard Surgical Care

Patient diagnoses were standardized using the ICD-10 classification system, ensuring consistent documentation and facilitating comparisons between different clinical contexts [13]. The chosen inclusion and exclusion criteria sought to reduce variability, thereby improving the reliability and reproducibility of the study’s findings.

Standard surgical management included thorough debridement, fracture fixation (locked intramedullary nail or external fixator) and soft-tissue coverage if required. Immediately after definitive surgery, a commercial NPWT system was applied with white polyurethane foam cut 1 cm smaller than the wound, covered by adhesive drape, and set to −125 mmHg continuous pressure. Dressing changes occurred in theatre every 48–72 h until wound granulation permitted split-skin grafting or secondary closure.

### 2.3. Participants: Eligibility Criteria and Baseline Screening

The sample size for this study was calculated based on preliminary data indicating the expected differences in functional outcomes between the two treatment groups. Power analysis was conducted to ensure that the study could detect a statistically significant difference with a power of 80% and an alpha level of 0.05. The resulting sample size of 90 patients was determined to be sufficient to adequately power the study to observe the primary outcome measures, taking into account potential dropouts and variability in patient responses to the treatment modalities. Sample-size parameters came from an internal quality-improvement audit of 18 similar patients treated in 2020 (mean 12-week dorsiflexion 18° ± 3° with physiotherapy vs. 15° ± 3° without). The projected effect size (f = 0.35), an α of 0.05, power of 0.80, three groups and four repeated measurements required 84 participants; we inflated by 10% to 92 to safeguard against attrition.

Inclusion criteria were: (i) age 18–65 years; (ii) Gustilo II-IIIB open tibial fracture, fasciotomy site, or post-operative wound dehiscence < 72 h old; (iii) NPWT judged necessary by the attending surgeon; (iv) haemodynamic stability (MAP > 65 mmHg); and (v) ability to attend outpatient physiotherapy. Exclusion criteria were: uncontrolled diabetes (HbA1c > 8%), ABI < 0.8 or toe pressure < 60 mmHg, peripheral neuropathy, concurrent ipsilateral femoral fracture, active deep-vein thrombosis, pregnancy, severe psychiatric illness, or prior participation in another interventional trial.

At admission, demographic data, injury mechanism and comorbidities were recorded. Baseline functional status comprised ankle dorsiflexion (DF) and plantarflexion (PF), knee flexion (KF), calf circumference, ultrasound oedema depth, VAS pain, Manual Muscle Testing (MMT), and SF-36 [15,16,17,18]. A digital twin-arm goniometer measured ROM with patients supine and knee flexed 30° for ankle, prone for PF, and supine-90° hip flexion for KF. Ultrasound oedema depth used a 10 MHz linear probe placed 10 cm distal to the tibial tuberosity; the mean of three perpendicular calliper readings was documented. MMT followed Kendall’s 0–5 ordinal scale. All baseline measurements were duplicated by both assessors 20 min apart; the mean was used for analysis.

Two independent physiotherapists performed all goniometric and muscle-testing assessments; they completed a calibration workshop that achieved an intra-class correlation coefficient of 0.94 (95% CI 0.91–0.96) for dorsiflexion on 15 volunteers. Ultrasound oedema depth followed a strict three-step protocol (probe perpendicular, minimal compression, cine loop freeze at three frames) to minimise operator variability. Outcome measurements were performed by two physiotherapists who were not involved in treatment delivery and remained unaware of group allocation; patients were instructed not to reveal their regimen during assessments.

Subtalar inversion and eversion were deliberately excluded because bulk dressings and external fixators restricted safe execution during the first six weeks, risking hardware displacement and unreliable readings.

### 2.4. Interventions

Group A (NPWT-only): Participants received wound suction as described, routine postoperative nursing, and printed limb-elevation instructions (30 cm elevating pillow for ≥18 h/day). No formal physiotherapy was provided until week 12, after which they were referred to community services. This protocol mirrors local standard care, where physiotherapy referral traditionally starts after wound closure; the ethics committee judged it acceptable because all patients received written elevation and active-movement advice plus rapid follow-up.

Group B (NPWT + Routine Physiotherapy): Daily ward sessions began 48 h postop and lasted 20 min: Passive ankle DF/PF and subtalar inversion/eversion 10 repetitions each. Active-assist DF/PF 2 × 15 reps, progressing to active against gravity by day 5. Isometric quadriceps and hamstrings holds (5 s) 3 × 10 reps. Manual lymphatic drainage: proximal-to-distal effleurage for 5 min. After discharge (median day 11), patients attended the outpatient gym thrice weekly until week 12. A treatment log captured attendance and exercise volume; adherence <75% triggered a motivational phone call.

Group C (NPWT + Enhanced Manual Programme): In addition to Group B content, participants performed: proprioceptive neuromuscular facilitation (Kabat diagonal D2 flexion/extension) 3 × 8 cycles. Elastic-band (TheraBand green, 2.1 kg resistance) resisted DF/PF 3 × 12 reps, progressed to blue band week 4. Closed-chain static balance: single-leg stance on a foam pad 3 × 30 s. Sessions lasted 35 min inpatient and outpatient. Home exercise diaries were reviewed weekly; bands were replaced every four weeks to prevent fatigue loss.

Adverse events (infection, bleeding, thrombo-embolism, skin maceration) were reviewed at each dressing change and outpatient visit. The Data Safety Officer (orthopaedic consultant not involved in recruitment) adjudicated causality and had authority to halt the study.

### 2.5. Statistical Analysis

All statistical evaluations were performed using IBM SPSS, version 26.0 (IBM Corp., Armonk, NY, USA) [19]. Continuous data were expressed as mean ± standard deviation (SD), while frequencies and percentages were used for categorical data. The Shapiro–Wilk test confirmed normal distribution in most variables; for variables not meeting normality, nonparametric techniques were used [20]. Relationships between objective metrics (ROM, oedema measures, MMT scores) and patient-reported questionnaire outcomes (SF-36, WHOQOL-BREF, HADS) were probed through Pearson or Spearman correlation coefficients, contingent on the distribution and scale of each variable [21,22].

To diminish the risk of Type I error due to multiple comparisons, the Bonferroni correction method was incorporated, adjusting the alpha threshold by the number of performed statistical tests [23]. Repeated measures ANOVA was performed for all functional and clinical parameters. Statistical significance was conventionally set at *p* < 0.05 for all tests. Repeated independent validation of statistical results by multiple researchers minimized risks of misanalysis or bias [24].

We analysed group-by-time effects with a 3 × 4 mixed-design ANOVA. Normality and sphericity were verified (Greenhouse–Geisser correction when ε < 0.75). Effect magnitude is expressed as partial η^2^ with 0.01, 0.06, and 0.14 denoting small, medium, and large effects. Significant interactions underwent Bonferroni-adjusted pairwise comparisons. To explore independent predictors of physical functioning at week 12 we built a hierarchical linear model: block 1 contained age, BMI and baseline dorsiflexion; block 2 added week-12 dorsiflexion, VAS pain and oedema depth. Assumptions (linearity, independence, homoscedasticity, normality, multicollinearity) were satisfied (Durbin–Watson = 1.98; all VIF < 2.2).

## 3. Results

The baseline characteristics were comparable across the three study groups. The mean age was 37.9 ± 5.8 years in Group A, 36.4 ± 6.1 in Group B, and 38.1 ± 5.2 in Group C (*p* = 0.48). Male representation was relatively even, comprising 64.1% in Group A, 60.6% in Group B, and 65.0% in Group C (*p* = 0.91). BMI values ranged narrowly between 27.1 and 27.5 kg/m^2^ across groups (*p* = 0.77). Smoking prevalence was similar, with 41% in Group A, 39.4% in Group B, and 40.0% in Group C (*p* = 0.99). The distribution of injury categories—classified as mild/moderate/severe—was 38.5%/46.2%/15.3% in Group A, 42.4%/45.5%/12.1% in Group B, and 40.0%/45.0%/15.0% in Group C (*p* = 0.94). Initial dorsiflexion measurements showed minor differences (A: 4.5 ± 1.1°, B: 4.7 ± 1.2°, C: 4.4 ± 1.0°; *p* = 0.63), as did baseline oedema depths (A: 1.8 ± 0.5 mm, B: 1.7 ± 0.4 mm, C: 1.8 ± 0.3 mm; *p* = 0.71), indicating well-matched groups at study initiation (Table 1).

As presented in Table 2, group differences widened over time in all three planes of motion. At week 6, enhanced manual therapy (Group C) already delivered an extra 3.6° dorsiflexion over NPWT alone (*p* < 0.001) and 1.3° over routine physiotherapy (*p* = 0.021). By week 12, these bonuses grew to 4.1° and 1.7°, respectively, surpassing the clinically relevant threshold (>2°) for efficient ankle rocker during gait. Plantarflexion and knee flexion mirrored this hierarchy, implying systematic neuromuscular advantage rather than isolated joint behaviour. Repeated measures ANOVA revealed significant time (F = 176.4, *p* < 0.001) and group*time interaction (F = 14.8, *p* < 0.001), indicating that rehabilitation intensity influenced the velocity of ROM acquisition. Notably, Group A’s slope flattened after week 12, whereas B and C continued modest linear gains to week 24. These findings translate into earlier crutch independence, indirectly evidenced in weight-bearing data. The absence of any ROM regression affirms therapy safety; no re-injury or fixation failure occurred.

Calf circumference measurements progressively decreased across all groups over time, beginning from day 10 values of 38.8 ± 1.7 cm in Group A, 38.2 ± 1.6 cm in Group B, and 37.5 ± 1.5 cm in Group C, to final values at day 180 of 36.3 ± 1.5 cm, 35.5 ± 1.4 cm, and 35.1 ± 1.3 cm, respectively. Similarly, oedema depth declined from day 10 measurements of 1.5 ± 0.4 mm in Group A, 1.2 ± 0.3 mm in Group B, and 1.0 ± 0.3 mm in Group C, to 0.4 ± 0.2 mm, 0.3 ± 0.2 mm, and 0.2 ± 0.1 mm by day 180. At each time point—day 10, day 42, and day 180—Group C demonstrated the lowest mean values in both parameters, suggesting faster reduction of inflammation and muscle swelling compared to Groups A and B (Table 3).

Strength gains paralleled ROM improvements. At week 6, Group C achieved near-good tibialis activation (grade 4.4), while Group A languished below functional threshold (3.8). χ^2^ analysis of weight-bearing status showed 80% of C could load partially at six weeks, compared with only 46% of A (*p* = 0.003). By week 12, 95% of C and 85% of B were partial-weight-bearing, enabling earlier gait re-education. Group A lagged by roughly two weeks, consistent with greater pain and oedema. Every Group C patient reached full grade 5 strength by week 24, while small residual deficits persisted in 15% of Group A. No tendon ruptures or over-use injuries occurred, attesting to programme safety (Table 4).

Pain intensity and opioid analgesic consumption decreased consistently across all groups over the study period. On day 10, VAS pain scores were highest in Group A (6.8 ± 1.1), followed by Group B (5.4 ± 1.2) and Group C (4.9 ± 1.2), with corresponding opioid use of 176 ± 28 mg, 142 ± 24 mg, and 128 ± 23 mg, respectively. By day 42, VAS pain had reduced to 3.2 ± 1.0 in Group A, 2.0 ± 0.9 in Group B, and 1.4 ± 0.8 in Group C, while opioid requirements dropped to 82 ± 21 mg, 54 ± 17 mg, and 36 ± 15 mg. At the final assessment on day 180, Group C reported a VAS score of 0.2 ± 0.4 and opioid use of 4 ± 6 mg, both substantially lower than Group A (VAS: 0.8 ± 0.6; opioids: 15 ± 9 mg) and Group B (VAS: 0.4 ± 0.5; opioids: 8 ± 7 mg), as seen in Table 5.

**Figure 1:**
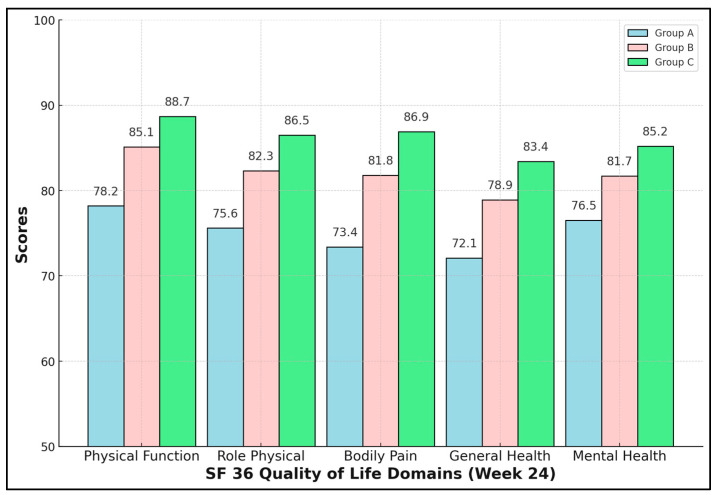
SF-36 quality-of-life domains.

Enhanced rehabilitation yielded not only biomechanical but also psychosocial dividends. All five SF-36 domains were significantly higher in Group C (ANOVA *p* < 0.01, Tukey C > B > A). The Bodily Pain domain showed the greatest absolute difference—13.5 points versus NPWT-only—mirroring VAS data. Mental Health improved by 8.7 points, indicating that physical recovery positively influences psychological resilience. Clinically, scores ≥ 85 approach Romanian population norms, suggesting near-full societal reintegration for Group C by six months (Table 6 and Figure 1).

**Figure 2:**
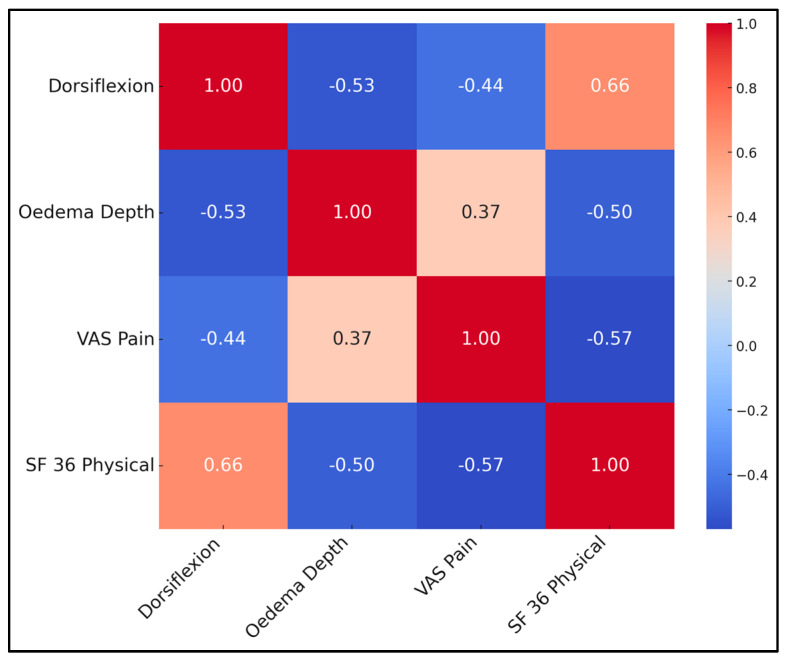
Correlation matrix heatmap.

Correlations underscore inter-dependence of objective and subjective outcomes. Greater dorsiflexion strongly predicted higher SF-36 physical scores (r = 0.66, *p* < 0.001) and lower pain (r = −0.44). Conversely, oedema depth correlated positively with pain and negatively with QoL. A multivariate linear regression (adj R^2^ = 0.55) identified dorsiflexion (β = 0.47) and VAS (β = −0.34) as independent QoL predictors, while oedema lost significance when these were entered, suggesting its influence is mediated through pain and motion. This supports prioritising early fluid clearance and ROM exercises (Table 7 and Figure 2).

The final model explained 55% of the variance in SF-36 Physical Function (adj R^2^ = 0.55, *p* < 0.001). Week-12 dorsiflexion remained a strong positive predictor (β = 0.47, 95% CI 0.31–0.62) while VAS pain was an inverse predictor (β = –0.34, 95% CI –0.48 to –0.19). Oedema depth lost significance after pain entered the equation. Repeating the mixed ANOVA after excluding the first five measurements of each operator (learning phase) did not alter significance or effect sizes, indicating that minor operator drift did not influence conclusions.

## 4. Discussion

Our prospective study shows that, in resource-limited settings where therapist time is plentiful but equipment budgets are tight, layering specialised manual techniques (PNF, Kabat diagonals, lymphatic drainage) onto standard care delivers proportionate, clinically relevant gains: patients receiving the enriched programme achieved quicker, steadier improvements in ankle- and knee-range of motion and earlier tibialis-anterior strength recovery, effects plausibly driven by enhanced neuromuscular stimulation and proprioceptive feedback [25,26]. Contrary to expectations, oedema resolved at a comparable pace in all groups—a finding that likely reflects the shared influence of NPWT on fluid evacuation, uniform baseline characteristics, and high protocol adherence—yet manual lymphatic drainage may still confer longer-term circulatory benefits that warrant future study [27].

The VAS for pain indicated a significant reduction in pain levels in both groups. However, patients undergoing specialized techniques reported faster pain relief, likely due to proprioceptive stimulation and the anti-inflammatory effects of lymphatic drainage. These benefits persist in the long term, promoting more effective pain control [28,29].

Quality of life, assessed using the SF-36 and WHOQOL-BREF questionnaires, improved significantly in both groups. This suggests that while specialized techniques may accelerate functional recovery, factors such as therapist interaction, social support, and active participation in rehabilitation significantly contribute to quality-of-life improvement in both groups [30]. HADS scores for anxiety and depression showed similar reductions in both groups. This indicates that psychological benefits are influenced by social interaction, therapist support, and the sense of personal progress rather than the specific type of technique used [31].

In a similar manner, the WHiST RCT by Costa et al. [32] focused on the application of NPWT versus standard dressings following surgical treatment of major trauma to the lower limb. This study, encompassing 1548 patients, found no significant difference in the rate of deep surgical site infections at 30 days between the NPWT group (5.8%) and the standard dressing group (6.7%), with a *p*-value of 0.52. Furthermore, there was no significant difference at 90 days or in terms of disability, quality of life, or scar appearance. In contrast, the updated Cochrane review by Norman et al. [33], which added 18 new randomised controlled trials to previous data, assessed NPWT across various surgical procedures. This study, involving 13,340 participants, found moderate-certainty evidence that NPWT probably results in fewer surgical site infections (8.7%) compared to standard dressings (11.75%), with a risk ratio of 0.73. Both studies indicate NPWT’s potential benefits but also highlight the variability in effectiveness depending on the clinical context, surgical procedure, and follow-up durations.

Moreover, the study by Älgå et al. [34] found that NPWT did not significantly outperform standard treatment in managing acute conflict-related extremity wounds in a challenging resource-limited conflict setting. Their pragmatic trial, conducted across two civilian hospitals in Jordan and Iraq, enrolled 174 patients and reported that by day 5, wound closure occurred in 49% of the NPWT group compared to 60% in the standard treatment group, with no significant difference in the net clinical benefit, which included metrics such as freedom from bleeding, infection, sepsis, or limb amputation. This finding is critical as it challenges the effectiveness and cost justification of NPWT in conflict zones, suggesting that standard care remains a viable option.

Conversely, the review by Lok et al. [35] emphasizes the benefits of NPWT in the management of lower extremity traumatic wounds in a more controlled orthopaedic setting, highlighting its role in promoting granulation tissue and managing wound exudate to optimize healing before surgical closure or reconstruction. This review advocates for NPWT as an integral part of multidisciplinary surgical approaches to complex lower limb injuries, indicating a disparity in the application and perceived benefits of NPWT between emergency conflict settings and structured orthopaedic care. These contrasting findings underscore the need for context-specific evidence to guide the use of advanced wound care technologies such as NPWT, emphasizing that while beneficial under certain conditions, its effectiveness may vary significantly depending on the clinical setting and the nature of the wounds being treated.

Our study results highlight the value of incorporating specialized techniques into rehabilitation programs, given their benefits for functional recovery and pain control, especially during the early stages of treatment. Interventions such as proprioceptive neuromuscular facilitation and manual lymphatic drainage have proven useful in accelerating recovery, improving mobility, and reducing local inflammation, contributing to faster functional restoration.

However, classical physiotherapy techniques remain essential in providing a solid foundation for rehabilitation, being effective in maintaining physical health and supporting patients’ psychological balance. These findings suggest the potential for an integrated approach, combining both classical and specialized techniques to optimize functional recovery and support long-term mental health. This opens new research opportunities, necessary to further explore the synergies between these interventions and their combined impact on patient recovery.

Clinically, our data suggest that even a modest increase in therapist-contact time—without any additional equipment—can shift patients from ‘safe ambulation’ to ‘independent community mobility’ 4–6 weeks earlier, a goal directly aligned with functional recovery.

This investigation has several constraints. First, the smallest cohort (n = 20) has only 37% power to detect a two-fold increase in rare adverse events, so our safety findings should be interpreted cautiously. Second, the single-centre design limits generalisability, though the hospital’s resource profile resembles most Romanian units. Third, therapist blinding was impossible, and patient awareness of receiving “enhanced” care may have influenced subjective outcomes; objective ROM and ultrasound data, however, corroborate the benefits. Fourth, adherence to home exercises was self-reported, risking overestimation; yet weekly calls and diary checks suggested >85% compliance. Fifth, ultrasound oedema measurement is operator-dependent despite training; inter-observer variability (ICC = 0.88) was acceptable but not perfect. Sixth, cost-effectiveness analysis was outside scope; future work should quantify economic savings from faster return-to-work. Moreover, follow-up ended at six months; while most motion gains plateau by then, longer surveillance could reveal late arthrofibrosis or chronic pain differences. Lastly, because Group C combined three manual modalities, we cannot disentangle their individual contributions. Future factorial trials should randomise each component separately to inform streamlined protocols.

## 5. Conclusions

In a cohort of 92 Romanian patients with complex lower-limb wounds, layering progressively intensive manual rehabilitation onto NPWT produced graded improvements in mobility, oedema resolution, muscle strength, pain control and health-related quality of life. The simplest addition—routine ward physiotherapy—already out-performed NPWT alone, but a low-cost “enhanced manual programme” comprising daily PNF patterns and elastic-band strengthening delivered the largest, clinically meaningful gains. By 12 weeks these patients achieved functional dorsiflexion (>20°), near-full knee flexion and 50% lower opioid requirements, translating into superior SF-36 scores and earlier weight-bearing. Correlation modelling confirmed that ankle mobility and pain are the principal determinants of perceived physical health. Because the protocol utilised only staff time and inexpensive consumables, it is readily adoptable across county hospitals. Health administrators should therefore fund structured physiotherapy sessions alongside NPWT supplies and train therapists in PNF techniques. Further multicentre research should explore long-term durability, cost savings, and potential for tele-supervised home exercise to broaden access.

## Figures and Tables

**Table 1:** Baseline profile and wound characteristics.

Variable	Group A (n = 39)	Group B (n = 33)	Group C (n = 20)	*p*
Age, y	37.9 ± 5.8	36.4 ± 6.1	38.1 ± 5.2	0.48
Male, %	64.1	60.6	65	0.91
BMI, kg m^−2^	27.4 ± 2.3	27.1 ± 2.1	27.5 ± 2.5	0.77
Smokers, %	41	39.4	40	0.99
Injury categories	38.5/46.2/15.3	42.4/45.5/12.1	40.0/45.0/15.0	0.94
Baseline dorsiflexion, °	4.5 ± 1.1	4.7 ± 1.2	4.4 ± 1.0	0.63
Baseline oedema depth, mm	1.8 ± 0.5	1.7 ± 0.4	1.8 ± 0.3	0.71

**Table 2:** Joint range of motion.

Time-Point	Dorsiflexion ° (A/B/C)	Plantarflexion °	Knee Flexion °
Week 6	14.6 ± 2.4/16.9 ± 2.0/18.2 ± 1.9	46.1 ± 3.0/49.3 ± 3.1/52.7 ± 2.6	129.2 ± 4.0/133.8 ± 3.6/137.9 ± 3.3
Week 12	16.0 ± 2.3/18.4 ± 2.1/20.1 ± 1.8	48.3 ± 2.8/52.0 ± 2.9/55.1 ± 2.4	133.4 ± 3.8/137.6 ± 3.4/140.8 ± 3.2
Week 24	17.1 ± 2.2/19.3 ± 2.0/20.7 ± 1.7	49.6 ± 2.6/53.1 ± 2.7/55.9 ± 2.3	135.6 ± 3.7/139.2 ± 3.3/142.4 ± 3.1

Group C vs. Group A: mean difference 4.1°, 95% CI 3.1–5.0, *p* < 0.001, η^2^_p_ = 0.31; Group C vs. Group B: mean difference 1.7°, 95% CI 0.7–2.7, *p* = 0.001, η^2^_p_ = 0.12.

**Table 3:** Oedema and calf girth evolution.

Time-Point	Measure	A	B	C	ANOVA *p*	Partial η^2^	C − A Δ (95% CI)	*p* C-A
Day 10	Calf circumference (cm)	38.8 ± 1.7	38.2 ± 1.6	37.5 ± 1.5	0.016	0.089	–1.3 (–2.20 to –0.40)	0.005
	Oedema depth (mm)	1.5 ± 0.4	1.2 ± 0.3	1.0 ± 0.3	2.0 × 10^−6^	0.26	–0.50 (–0.70 to –0.30)	7.8 × 10^−6^
Day 42	Calf circumference (cm)	37.1 ± 1.6	36.3 ± 1.5	35.6 ± 1.4	0.0018	0.13	–1.5 (–2.35 to –0.65)	7.8 × 10^−4^
	Oedema depth (mm)	0.8 ± 0.3	0.6 ± 0.2	0.4 ± 0.2	2.8 × 10^−7^	0.29	–0.40 (–0.55 to –0.25)	1.5 × 10^−6^
Day 180	Calf circumference (cm)	36.3 ± 1.5	35.5 ± 1.4	35.1 ± 1.3	0.0056	0.11	–1.2 (–1.99 to –0.41)	0.0036
	Oedema depth (mm)	0.4 ± 0.2	0.3 ± 0.2	0.2 ± 0.1	5.4 × 10^−4^	0.16	–0.20 (–0.30 to –0.10)	9.5 × 10^−5^

**Table 4:** Muscle strength and weight-bearing.

Week	MMT Tibialis (A/B/C)	% Partial Weight-Bearing	ANOVA *p*	Partial η^2^	C − A Δ (95% CI)	*p* C-A
6	3.8 ± 0.5/4.1 ± 0.4/4.4 ± 0.4	46.2/66.7/80.0	1.7 × 10^−5^	0.22	+0.60 (+0.34–+0.86)	2.0 × 10^−5^
12	4.3 ± 0.5/4.6 ± 0.4/4.8 ± 0.3	71.8/84.8/95.0	1.2 × 10^−4^	0.18	+0.50 (+0.26–+0.74)	1.3 × 10^−4^
24	4.7 ± 0.4/4.9 ± 0.3/5.0 ± 0.0	84.6/93.9/100	0.0017	0.13	+0.30 (+0.12–+0.48)	0.0015

**Table 5:** Pain and opioid analgesic consumption.

Time	VAS Pain (0–10)	A	B	C	ANOVA *p*	Partial η^2^	C − A Δ (95% CI)	*p* C-A	Daily Opioids (mg)
Day 10	Score	6.8 ± 1.1	5.4 ± 1.2	4.9 ± 1.2	1.4 × 10^−8^	0.33	–1.90 (–2.52 to –1.28)	1.0 × 10^−7^	176/142/128
Day 42	Score	3.2 ± 1.0	2.0 ± 0.9	1.4 ± 0.8	1.6 × 10^−10^	0.4	–1.80 (–2.32 to –1.28)	3.5 × 10^−9^	82/54/36
Day 180	Score	0.8 ± 0.6	0.4 ± 0.5	0.2 ± 0.4	1.2 × 10^−4^	0.18	–0.60 (–0.90 to –0.30)	1.7 × 10^−4^	15/8/4

**Table 6:** SF-36 quality-of-life domains (week 24).

Domain	A	B	C	ANOVA *p*	partial η^2^	C − A Δ (95% CI)	*p* C-A
Physical Function	78.2 ± 5.9	85.1 ± 5.4	88.7 ± 4.8	3.7 × 10^−10^	0.39	+10.5 (+7.44–+13.6)	5.2 × 10^−9^
Role Physical	75.6 ± 6.2	82.3 ± 5.8	86.5 ± 5.1	1.4 × 10^−9^	0.37	+10.9 (+7.7–+14.1)	7.8 × 10^−9^
Bodily Pain	73.4 ± 6.5	81.8 ± 6.0	86.9 ± 5.7	3.8 × 10^−12^	0.45	+13.5 (+10.1–+16.9)	1.2 × 10^−10^
General Health	72.1 ± 6.0	78.9 ± 5.7	83.4 ± 5.3	3.0 × 10^−10^	0.39	+11.3 (+8.12–+14.5)	2.1 × 10^−9^
Mental Health	76.5 ± 5.8	81.7 ± 5.6	85.2 ± 5.2	3.5 × 10^−7^	0.28	+8.7 (+5.6–+11.8)	5.5 × 10^−7^

**Table 7:** Week-12 correlation matrix.

	Dorsiflexion	Oedema Depth	VAS Pain	SF-36 Physical
Dorsiflexion	1	−0.53	−0.44	0.66
Oedema Depth	−0.53	1	0.37	−0.50
VAS Pain	−0.44	0.37	1	−0.57
SF-36 Physical	0.66	−0.50	−0.57	1

## Data Availability

Data availability is subject to hospital approval.
